# Neither dynamic, static, nor volumetric variables can accurately predict fluid responsiveness early after abdominothoracic esophagectomy

**DOI:** 10.1186/2047-0525-2-3

**Published:** 2013-02-22

**Authors:** Hironori Ishihara, Eiji Hashiba, Hirobumi Okawa, Junichi Saito, Toshinori Kasai, Toshihito Tsubo

**Affiliations:** 1 Department of Anesthesiology, Hirosaki University Graduate School of Medicine, 5 Zaifu-Cho, Hirosaki-Shi 036-8562, Japan

**Keywords:** Cardiac preload, Esophagectomy, Fluid responsiveness, Glucose, Intrathoracic blood volume, Stroke volume variation

## Abstract

**Background:**

Hypotension is common in the early postoperative stages after abdominothoracic esophagectomy for esophageal cancer. We examined the ability of stroke volume variation (SVV), pulse pressure variation (PPV), central venous pressure (CVP), intrathoracic blood volume (ITBV), and initial distribution volume of glucose (IDVG) to predict fluid responsiveness soon after esophagectomy under mechanical ventilation (tidal volume >8 mL/kg) without spontaneous respiratory activity.

**Methods:**

Forty-three consecutive non-arrhythmic patients undergoing abdominothoracic esophagectomy were studied. SVV, PPV, cardiac index (CI), and indexed ITBV (ITBVI) were postoperatively measured by single transpulmonary thermodilution (PiCCO system) after patient admission to the intensive care unit (ICU) on the operative day. Indexed IDVG (IDVGI) was then determined using the incremental plasma glucose concentration 3 min after the intravenous administration of 5 g glucose. Fluid responsiveness was defined by an increase in CI >15% compared with pre-loading CI following fluid volume loading with 250 mL of 10% low molecular weight dextran.

**Results:**

Twenty-three patients were responsive to fluids while 20 were not. The area under the receiver-operating characteristic (ROC) curve was the highest for CVP (0.690) and the lowest for ITBVI (0.584), but there was no statistical difference between tested variables. Pre-loading IDVGI (r = −0.523, *P* <0.001), SVV (r = 0.348, *P* = 0.026) and CVP (r = −0.307, *P* = 0.046), but not PPV or ITBVI, were correlated with a percentage increase in CI after fluid volume loading.

**Conclusions:**

These results suggest that none of the tested variables can accurately predict fluid responsiveness early after abdominothoracic esophagectomy.

## Background

Abdominothoracic esophagectomy for esophageal cancer is a major surgical procedure with high rates of morbidity and mortality [1]. According to our experience, approximately 60% of patients who undergo esophagectomy develop hypotension requiring subsequent fluid volume loading during the first 15-h postoperative period, even though cardiovascular states immediately after surgery are relatively stable and/or postoperative bloody drainage is minimal [2].

Over the last decade, respiratory variations or dynamic preload variables such as stroke volume variation (SVV) and pulse pressure variation (PPV) have been reported to be better predictors of fluid responsiveness than commonly monitored static preload variables such as cardiac filling pressures [3,4], even though SVV or PPV measurements generally require mechanical ventilation (tidal volume >8 mL/kg) in the absence of spontaneous breathing and/or cardiac arrhythmias [4,5]. However, Cannesson *et al*. [6] recently showed that approximately 25% of patients undergoing prediction of fluid responsiveness are in the ‘grey zone’ of PPV and that a ‘black-or-white’ decision based on the receiver-operating characteristic (ROC) curve approach does not fit the reality of clinical or screening practice.

To our knowledge, only one study has measured SVV after esophagectomy, suggesting that SVV is clinically relevant as a guide of fluid volume management [7]. However, as this measurement was made in the presence of spontaneous respiratory activity (pressure support ventilation), it would be of limited use in evaluating fluid responsiveness. Abdominothoracic esophagectomy can lead to hemodynamic instability soon after the operation [8] and may also modify the original thoracic structure, decreasing the constraints of the chest wall imposed on the heart and the lungs and altering cyclic changes in intrathoracic pressure on heart-lung interactions. We therefore hypothesized that studies into fluid responsiveness would be of limited value during such hemodynamically unstable states.

The initial distribution volume of glucose (IDVG) has been proposed as a representative of the central extracellular fluid (ECF) volume status without significant modification of glucose metabolism [9,10], and can be measured simply and rapidly in any intensive care unit (ICU) by injecting a small amount of glucose (5 g) and determining plasma glucose changes 3 min post injection [11]. Measurements can also be repeated at 30-min intervals without sustained increases in plasma glucose [12]. We previously reported that IDVG, rather than intrathoracic blood volume (ITBV) or central venous pressure (CVP), is closely correlated with cardiac output (CO) during hypotension and subsequent fluid volume loading early after esophagectomy [8]. Moreover, IDVG was recently reported to predict hypovolemic hypotension early after abdominal aortic surgery [13] and to have an inverse correlation with PPV after the induction of anesthesia in neurosurgical patients [14]. Accordingly, IDVG has the potential to be a useful marker of fluid responsiveness.

This study aimed to evaluate the ability of currently available preload variables such as SVV, PPV, CVP, and ITBV as well as IDVG to predict fluid responsiveness early after admission to the ICU following abdominothoracic esophagectomy.

## Methods

Ethical approval for this study was provided by the Ethical Committee of Hirosaki University Graduate School of Medicine, Hirosaki, Japan, and each patient gave written informed consent before surgery. The study was planned to consist of at least 31 hypotensive patients, since a sample size of 31 is required to detect differences of 0.10 between areas under the ROC curve (5% type I error rate, 80% power, two-tailed test) [15]. Patients with aortic aneurysms and/or sustained arrhythmias including atrial fibrillation were excluded from the study. Those with diabetes mellitus and/or cardiovascular diseases such as hypertension without apparent ischemic heart disease were included. Preoperative echocardiographic measurements of left ventricular ejection fraction were required to exceed 60%.

Each patient underwent radical surgery for esophageal cancer that was performed using a right thoracoabdominal approach together with extensive resection of adjacent lymph nodes, subcarinal lymph nodes and/or cervical lymph nodes. Fluid and cardiovascular management decisions during anesthesia, including amounts of crystalloid solution and use of colloidal solutions, blood products or vasoactive drugs, were made by individual anesthesiologists. No patients received a continuous infusion of vasoactive drugs during anesthesia. Neither vasoactive drugs nor blood products were administered when patients arrived at the ICU. All patients postoperatively received controlled mechanical ventilation (tidal volume >8 mL/kg of ideal body weight), with peak airway pressure above positive end-expiratory pressure (10–15 cmH_2_O, respiratory rate 12-15/min) with a low positive end-expiratory pressure (<5 cm H_2_O) and continuous infusions of propofol (2–3 mg/kg/h) and morphine (0.4-0.8 mg/h) at least until the completion of the study. Supplemental midazolam (2–6 mg/h) was infused to achieve complete control of ventilation without spontaneous respiration during the study period. The infusion rate of these sedatives and analgesics was kept constant from at least 30 min before and during the study period. Although each patient had a thoracic epidural catheter for postoperative analgesia, epidural analgesia was only started after completion of the study.

Both 4.3% glucose solution with electrolytes and lactated Ringer’s solution were infused simultaneously at a constant rate of 1.5 mL/kg/h and 1.0 mL/kg/h, respectively, for at least 12 h after surgery. No vasoactive drugs were administered throughout the study period. One patient required continuous infusion of insulin (1 U/h) throughout the study period.

### Measurement of fluid volume loading

Measurements were made twice: the first was taken during the 90 min after postoperative admission to the ICU on the operative day in the absence of apparent hypotension (pre-loading) followed by fluid volume loading with 250 mL of 10% low molecular weight dextran 40 (Otsuka Pharmaceutical Factory Inc., Tokyo, Japan) over a period of 20 min. The second measurement was made 10 min after completion of fluid volume loading (post- loading).

A right subclavian venous catheter had been put in place before the operative day. A thermistor tipped catheter for thermodilution and pulse contour analysis (PV2015L20N, Pulsion, Munich, Germany) was inserted into a femoral artery and connected to the PiCCO monitoring system (PiCCO plus, Pulsion) in the ICU immediately after surgery as described elsewhere [16]. Transducers monitoring arterial pressure and CVP were positioned at the mid-axillary level with atmospheric pressure used as the zero reference level. Ten mL of cold isotonic saline solution (<8°C) was injected through the right subclavian venous line to determine CO and ITBV before and after fluid volume loading. A variation of ±10% within triplicate measurements of CO was defined as acceptable. Coefficients of variation for repeated CO measurements were ≤7.3%. SVV and PPV were recorded automatically as percentage changes using the PiCCO monitoring system [16].

Immediately after these measurements were taken, 10 mL of 50% glucose solution (Otsuka Pharmaceutical Factory Inc.) (5 g) was injected through the same central venous line to calculate the IDVG. Blood samples were obtained through a radial artery catheter immediately before and 3 min after the injection. Plasma was separated immediately, and measurements of glucose concentrations were performed within 5 min of sampling. Plasma glucose concentrations of all blood samples were measured using amperometry by a glucose oxidase immobilized membrane-H_2_O_2_ electrode (glucose analyzer GA-1150; Arkray Co, Ltd, Kyoto, Japan). IDVG was calculated using the difference between plasma glucose concentrations immediately before and 3 min after the glucose injection as described previously [11]. Measurements were made in duplicate. Coefficients of variation for repeated measurements were 1.0% or less for plasma glucose (range, 3.0-17.0 mmol/L). Routine hemodynamic and clinical variables including automated SVV and PPV were recorded immediately before each volume measurement.

### Statistical analysis

Unless otherwise stated, data are presented as mean (SD) and median (interquartile range) values. ITBV, stroke volume, CO, and IDVG are indexed to body surface area based on the reported preoperative height and body weight. Statistical analysis was performed with SigmaPlot 12 (Systat Software Inc., San Jose, CA, USA). Pre- and post-volume loading variables were compared using a paired *t*-test for normally distributed data. The Wilcoxon signed rank test was used for data that were not normally distributed.

Fluid responsiveness was defined by an increase in cardiac index (CI) >15% as reported previously [17,18], since most reports using the percentage increase in CO or stroke volume (SV) for this purpose set the threshold value at 10% or 15% after a 250 or 500 mL fluid challenge [4]. The ability to predict fluid responsiveness was quantified for each preload variable by calculating the area under the ROC curve. The diagnostic value was defined from this value as: excellent, >0.85; good, >0.75; and poor 0.50-0.75 [19]. The cutoff value was chosen to minimize the mathematical distance to the ideal point (sensitivity = specificity = 1). Pearson’s linear correlation was performed to determine the relationship between each preloading variable and the percentage changes in CI after fluid volume loading (△CI), between each actual preloading and postloading variable and the corresponding CI, and between changes in each preloading and postloading variable and those in CI. *P* <0.05 was considered significant.

## Results

Initially, 45 consecutive patients with no arrhythmias after esophagectomy were recruited to the study. Of these, two were excluded because of technical problems in measuring CO, and the development of hypotension soon after admission to the ICU, requiring fluid volume loading during the first measurement. Thus, 43 consecutive patients were finally enrolled into the study. Of these, 23 patients were responsive to fluids and 20 were not.

Table 1 shows patient demographics and fluid management during anesthesia and surgery. Only the amounts of administered lactated Ringer’s solution and estimated intraoperative blood loss different between the groups (*P* = 0.030, respectively). During the study period, two patients had apparent continuous air leakage from the chest drainage tube so their SVV and PPV data were excluded to prevent inaccuracies [20]. Table 2 shows the comparison of cardiovascular variables and tidal volume between responders and non-responders. Only pre-loading CVP and IDVGI differed between these two groups (*P* = 0.033 and *P* = 0.043, respectively), and the remaining variables showed no differences (Table 3).

**Table 1:** Patients demographics and fluid management during anesthesia and surgery

	**Responders (*n *** **= 23)**	**Non-responders (*n *** **= 20)**	**P**^**z**^
Gender (Male/Female)	22/1	20/0	1.000
Age (years)	65 ± 6 (66, 60–69)	65 ± 7 (65, 61–70)	0.815
Height (m)	1.65 ± 0.05 (1.67, 1.63-1.70)	1.66 ± 0.05 (1.67, 1.63-1.70)	0.279
Preoperative body weight (kg)	60.9 ± 8.6 (60.9, 56.9-64.8)	59.8 ± 7.9 (60.0, 54.1-67.6)	0.702
Body surface area (m^2^)	1.67 ± 0.12 (1.68, 1.59-1.74)	1.67 ± 0.11(1.68, 1.60-1.75)	0.970
Duration of surgery (hrs)	6.9 ± 1.3, (6.6, 6.3- 7.6)	7.3 ± 1.1, (7.2, 6.8- 7.8)	0.090
Lactated Ringer’s solution (L)	4.6 ± 1.0 (4.5, 3.9-5.0)	5.4 ± 1.3 (5.6, 4.5-6.0)	0.030
Patients receiving packed red cell (*n*)	5 (260–520)	3 (260–780)	0.704
Patients receiving colloids (*n*)	8 (250–750)	7 (250–1320)	1.000
Urine output (mL)	600 ± 390 (410, 310–770)	610 ± 390 (480, 330–790)	0.808
Estimated blood loss (g)	750 ± 400 (650, 470–1000)	810 ± 490 (680, 520–900)	0.030

Data are presented as mean ± SD (median, interquartile range) or as number of patients (range of administered volume).

^a^Between responders and non-responders.

**Table 2:** Comparison of routine cardiovascular variables and tidal volume between responders and non-responders

		**Responders**	**Non-responders**	**P**^**a**^
Heart rate (bpm)	Pre	74 ± 13 (73, 66–80)	69 ± 11 (69, 59–77)	0.143
	Post	75 ± 12 (73, 65–80)	69 ± 9 (68, 62–77)	0.066
Mean arterial pressure (mmHg)	Pre	82 ± 14 (82, 70–88)	86 ± 16 (83, 75–95)	0.301
	Post	93 ± 15 (90, 83–105)^b^	93 ± 14 (93, 82–100)^c^	0.91
Cardiac index (L/min/m^2^)	Pre	2.6 ± 0.5 (2.6, 2.2-3.0)	2.9 ± 0.3 (2.9, 2.7-3.1)	0.063
	Post	3.4 ± 0.6 (3.4, 2.9-3.8)^b^	3.1 ± 0.3 (3.1, 2.9-3.4)^b^	0.095
Stoke volume index (mL/m^2^)	Pre	36.4 ± 7.7 (34.5, 31.2-42.4)	42.5 ± 5.4 (42.8,38.6-47.5)	0.005
	Post	46.4 ± 7.6 (47.3, 39.8-52.2)^b^	45.6 ± 4.3 (46.4, 42.2-48.9)^b^	0.635
Hematocrit (%)	Pre	30.9 ± 4.4 (29.5, 27.4-32.7)	30.4 ± 4.0 (29.8, 27.7-32.1)	0.855
	Post	27.5 ± 4.1 (26.7, 24.5-29.6)^b^	27.9 ± 4.1 (27.6, 25.5-29.8)^b^	0.661
Tidal volume (mL/kg)^d^		9.0 ± 1.0 (8.8, 8.3-9.4)	8.8 ± 0.8 (8.5, 8.1-9.3)	0.342

Data are presented as mean ± SD (median, interquartile range).

^a^Between responders and non-responders.

^b^*P* <0.001 as compared with pre-fluid loading.

^c^*P *<0.05.

^d^Per kg of ideal body weight.

Pre, Immediately before volume loading; Post, 10 min after volume loading.

**Table 3:** Comparison of cardiac preload variables between responders and non-responders

		**Responders**	**Non-responders**	***P***^**a**^
Stroke volume variation (%)	Pre	13.9 ± 4.6 (13.0, 11.0-16.3)	12.2 ± 4.1 (11.5, 9.0-15.5)	0.232
	Post	7.8 ± 4.1 (7.0, 4.8-10.3)^b^	6.4 ± 2.2 (6.0, 5.0-7.0)^b^	0.27
Pulse pressure variation (%)	Pre	10.6 ± 3.5 (9.0, 8.0-13.0)	8.7 ± 3.7 (8.5, 6.0-11.0)	0.107
	Post	4.9 ± 2.4 (4.0, 3.5-5.0)^b^	4.5 ± 1.4 (4.0, 3.5-5.0)^b^	0.862
Central venous pressure (mmHg)	Pre	5 ± 2 (5, 4–7)	7 ± 3 (7, 5–9)	0.033
	Post	7 ± 2 (8, 6–9)^b^	9 ± 2 (9, 8–11)^b^	0.008
ITBVI(L/m^2^)	Pre	0.78 ± 0.1 (0.78, 0.72-0.85)	0.81 ± 0.09 (0.83, 0.75-0.87)	0.292
	Post	0.84 ± 0.09 (0.85, 0.77-0.91)^b^	0.84 ± 0.10 (0.84, 0.76-0.89)	0.964
IDVGI(L/m^2^)	Pre	4.2 ± 0.7 (4.2, 3.9-4.8)	4.6 ± 0.5 (4.6, 4.3-4.8)	0.043
	Post	4.8 ± 0.7 (4.9, 4.4-5.3)^b^	4.9 ± 0.6 (5.0, 4.8-5.2)^b^	0.574

Data are presented as mean ± SD (median, interquartile range).

^a^Between responders and non-responders.

^b^*P *<0.001 as compared with pre-fluid volume loading values.

ITBVI, Indexed intrathoraci blood volume; IDVGI, Indexed initial distribution volume of glucose; Pre, Immediately before volume loading; Post, 10 min after volume loading.

**Figure 1:**
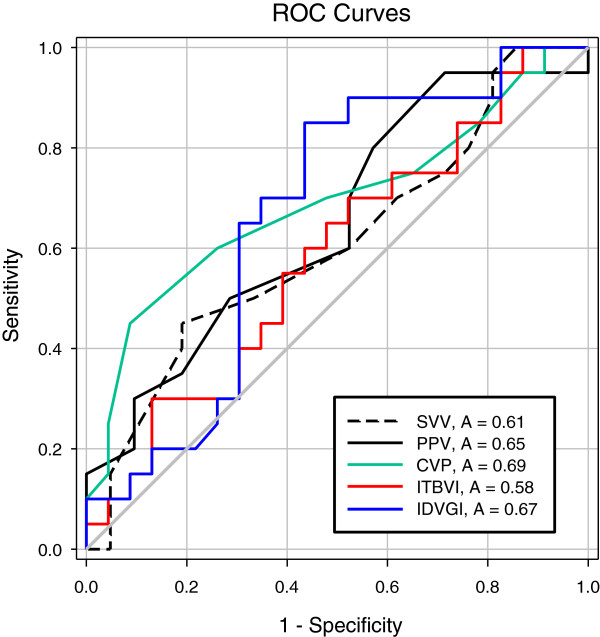
**Receiver-operating characteristic (ROC) curves comparing the ability of various preload variables to discriminate responders and non-responders. **CVP, Central venous pressure; IDVGI, Indexed initial distribution volume of glucose; ITBVI, Indexed intrathoracic blood volume; PPV, Pulse pressure variation; SVV, Stroke volume variation.

**Figure 2:**
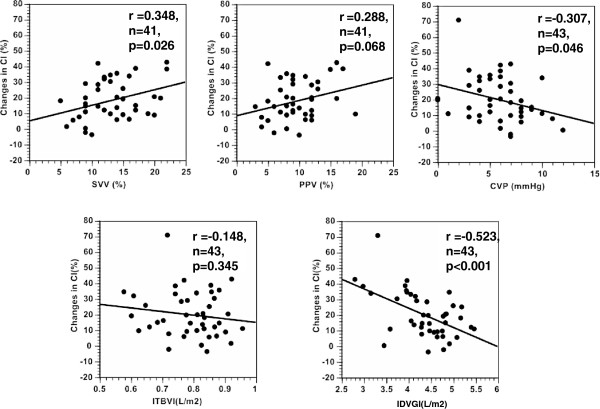
**The relationship between each cardiac preload variable before volume loading and the percent change of cardiac index after volume loading. **Top left: SVV, Stroke volume variation; Top middle: PPV, Pulse pressure variation; Top right: CVP, Central venous pressure; Bottom left: CI, Cardiac index; IDVGI, Indexed initial distribution volume of glucose; ITBVI, Indexed intrathoracic blood volume.

The area under the ROC curve for evaluation of the ability to predict fluid responsiveness was highest for CVP (0.690) and lowest for ITBVI (0.584), but this difference was not statistically significant (Figure 1, Table 4). Pre-loading IDVGI was inversely correlated with △CI (r = −0.523, *P* <0.001), and pre-loading SVV and CVP were slightly correlated with △CI (r = 0.348, *P* = 0.026 and r = −0.307, *P* = 0.046, respectively) (Figure 2). Neither preloading PPV nor ITBVI were correlated with △CI (r = 0.288, *P* = 0.068 and r = −0.148, *P* = 0.345, respectively).

**Table 4:** Diagnostic parameters for predicting fluid responsiveness

	**Area under ROC curve**	**95% CI**	**Best threshold**	**Sensitivity (%)**	**Specificity (%)**	**PPV (%)**	**NPV (%)**
SVV (%)	0.609	0.433-0.785	10.5	45	81	70	60
PPVa (%)	0.651	0.481-0.821	8.5	50	71	64	59
CVP (mmHg)	0.69	0.524-0.856	6.5	60	74	70	65
ITBVI (L/m^2^)	0.584	0.410-0.758	0.78	65	48	55	58
IDVGI (L/m^2^)	0.666	0.497-0.835	4.23	85	57	66	79

CI, Confidence interval; CVP, Central venous pressure; IDVGI, Indexed initial distribution volume of glucose; ITBVI, Indexed intrathoracic blood volume; NPV, Negative predictive value; PPV, Positive predictive value; PPVa, Pulse pressure variation; SVV, Stroke volume variation.

Using all data before and after fluid volume loading, actual or changed IDVGI showed the highest correlation with actual and changed CI, respectively (Table 5).

**Table 5:** Correlation coefficient with cardiac index using pre-and post-fluid volume loading data

	**Actual values (r)**	**Changed values (r)**
	**(*n *** **= 86)**	**(*n *** **= 43)**
SVV (%)	−0.367 (*P *<0.001)^a^	−0.0445 (*P *= 0.782)^b^
PPV (%)	−0.414 (*P *<0.001)^a^	−0.0158 (*P *= 0.922)^b^
CVP (mmHg)	0.320 (*P *= 0.003)	−0.0530 (*P *= 0.736)
ITBVI (L/m^2^)	0.335 (*P *= 0.002)	0.215 (*P *= 0.165)
IDVGI (L/m^2^)	0.561 (*P *<0.001)	0.473 (*P *= 0.001)

^a^*n *= 82.

^b^*n *= 41.

CVP, Central venous pressure; IDVGI, Indexed initial distribution volume of glucose; ITBVI, Indexed intrathoracic blood volume; PPV, Pulse pressure variation; SVV, Stroke volume variation.

## Discussion

Most fluid responsiveness studies have been performed in the absence of severe hypotension. For ethical reasons, however, it is not appropriate to give fluid volume loading to normotensive patients, particularly non-responders in this study who received a large fluid volume during anesthesia and surgery, unless some indication is present.

Major surgical procedures result in a shift of the ECF from the central to the peripheral compartment as well as generalized capillary protein and water leakage both intra- and postoperatively [21]. Indeed, two patients in the present study recorded decreased CI despite fluid volume loading. Furthermore, most patients in this study required additional volume loading and/or an infusion of noradrenaline to overcome repeated hypotension throughout the first postoperative day. As hypotension development can be affected by hypovolemia and changes in peripheral vascular resistance, we believe that our study is ethically appropriate even in the absence of severe hypotension early after abdominothoracic esophagectomy.

The present study demonstrates that none of the tested variables can accurately predict fluid responsiveness soon after abdominothoracic esophagectomy, as assessed by the area under the ROC curve, even though definitions of fluid responsiveness may have a major impact on the results of SVV or PPV validity [22]. Fluid responders in this study were defined by an increase in CI >15% after fluid volume loading as reported previously [17,18]. When CI or stroke volume index (SVI) for evaluating fluid responsiveness set the threshold value at 10%, the number of fluid responders and non-responders differed (31 *versus* 12 for CI at 10%, and 33 *versus* 10 for SVI at 10%) suggesting that ROC curve analysis is inadequate since it assumes an expected rate of fluid responsiveness of 50% [6,23]. When the threshold value for SVI was set at 15%, only SVV reached the lowest edge of a ‘good’ diagnostic value (0.757), but there was no statistical difference among tested variables indicating no obvious difference from the present results(Additional file 1: Figure S1). Accordingly, we believe that a threshold value of 15% for CI in this study was adequate, even though the ‘grey zone’ approach has been proposed to avoid the binary constraints of a ‘black-or-white’ decision of the ROC curve approach [6].

The open-chest surgical procedure was performed with a right thoracoabdominal approach and it is likely that the extensive resection of adjacent lymph nodes, subcarinal lymph nodes, cervical lymph nodes and esophageal substitution such as gastric advancement would modify the intrathoracic structure. Furthermore, postoperative left pleural effusion was common. Two of the 43 patients experienced continuous air leakage from the chest drainage tube, so were excluded from the SVV and PPV study to avoid potential inaccuracies [20]. Indeed, these two patients had a low SVV (both 7%) and PPV (11% and 7%), despite an obvious increase in △CI (71% and 34%). Although a closed-chest condition after open-chest coronary artery bypass graft surgery enables the assessment of fluid responsiveness [24], changes in these thoracic structures and reduction of the pericardial constraint may have abated the effects of cyclic changes in intrathoracic pressure to heart-lung interactions even in the absence of an open-chest condition after esophagectomy. Such pathophysiology may lead to inaccuracies in respiratory variation results.

Of the variables tested, only pre-loading IDVGI had an inverse correlation with △CI following fluid volume loading in this study (power = 0.956). We used 250 mL of 10% dextran 40 solution for fluid volume loading, which has an oncotic pressure of 40 mmHg. Subsequent increments in plasma volume can exceed its infusion volume by up to 1.5 times while depleting the interstitial fluid volume [25], which could have an important impact on IDVG after fluid volume loading since IDVG represents both intravascular volume and the interstitial fluid volume of highly perfused tissues [10]. An inconsistent increase in IDVG associated with unchanged mean arterial pressure after colloid loading has also been reported following cardiac surgery [26]. Additionally, Harvey *et al*. [27] showed that IDVG and systolic area variability could not predict fluid responsiveness following cardiac surgery. Presumably, the presence of hemodynamically unstable states caused by internal bleeding, temperature change, alteration in vasomotor tone, or fluid shifts between compartments during volume loading rather than methodological flaws of IDVG would also play a role in these inconsistent results [28].

Although this study showed the limited predictive value of tested variables for fluid responsiveness, measurement of IDVG is desirable when either hypotension or decreases in arterial blood pressure occur early after abdominothoracic esophagectomy since IDVG has the highest correlation with CO, as reported previously [8]. When a small IDVG (<110 mL/kg) is observed, fluid volume loading is indicated and *vice versa*. However, an infusion of noradrenaline is indicated when a large IDVG (>130 mL/kg) is observed.

The present study has a number of limitations. First, most studies on fluid responsiveness evaluated post-fluid volume loading variables at the completion of fluid volume loading or soon after its completion [6,18,29]. In this study, however, its measurement was performed 10 min after completion of fluid volume loading as reported previously [8], since a minimum interval of 30 min is required for repeated IDVG measurements to avoid sustained hyperglycemia [12]. Consequently, many of the important signals may have been lost during this 10-min period, particularly during hemodynamic unstable states. However, the magnitude of an increase in CI after fluid volume loading in fluid responders of this study was comparable or even greater than other fluid responsive studies [6,30], supporting the idea that the poor predictive values of tested variables were not attributable to insufficient signals in post-fluid loading measurements.

Second, we used 10% low molecular weighted dextran for fluid volume loading as reported previously [8], although 6% hydroxyethyl starch (medium molecular weight: 200,000 Da) is now widely used for this purpose and may have been more desirable in our study. However, considering the almost equivalent effects of either colloid on plasma volume expansion [25], it is likely that we can extrapolate our results to studies using 6% hydroxyethyl starch.

Third, we administered a fixed amount of dextran rather than an amount based on body weight. However, as determined by preoperative body weight, its variability was lower than that of other fluid responsiveness studies [6,20,23], reflecting insufficient preoperative nutritional status. As a lower preoperative body weight was observed in the majority of patients prior to diagnosis of esophageal cancer, most were administered approximately 4 mL/kg dextran.

Fourth, we did not test fluid responsiveness in the presence of hypotension or cardiac compromised conditions. Nevertheless, preload variables might be useful during hemodynamically unstable states such as hypotension when either fluid volume loading or an infusion of vasoactive drugs is required. Further studies are therefore required to assess this. Finally, we did not evaluate simultaneous echocardiography so an adequate view of cardiac chambers could not be consistently obtained following surgery for esophageal cancer.

## Conclusions

Our data demonstrate that none of the dynamic, static, or volumetric variables measured in this study can be accurately used as a predictor of fluid volume responsiveness early after abdominothoracic esophagectomy.

## Abbreviations

CI: Cardiac index; CO: Cardiac output; CVP: Central venous pressure; ECF: Extracellular fluid; GEDV: Global end-diastolic volume; IDVG: Initial distribution volume of glucose; ITBV: Intrathoracic blood volume; MTT: Mean transit time; PEEP: Positive end-expiratory pressure; PPV: Pulse pressure variation; ROC: Receiver operating characteristic; SV: Stroke volume; SVI: Stroke volume index; SVV: Stroke volume variation

## Competing interests

The authors declare that they have no competing interests.

## Authors’ contributions

HI conceived of the study, designed the study, carried out data collection, statistical analysis, and drafted the manuscript. EH participated in its design and helped to draft the manuscript. JS, TK, HO, and TT participated in the data collection from the patients. All authors read and approved the final manuscript.

## Supplementary Material

Additional file 1: Figure S1 Fluid responsiveness was defined by an increase in stroke volume index (SVI) >15% after volume loading compare to the pre-loading SVI. Click here for file
