# Supplementary material for: Neither dynamic, static, nor volumetric variables can accurately predict fluid responsiveness early after abdominothoracic esophagectomy
Source: Perioper Med (Lond). 2013 Feb 22;2:3. doi: 10.1186/2047-0525-2-3 (PMC3964321; doi:10.1186/2047-0525-2-3)
Supplement: Additional file 1: Figure S1 — Fluid responsiveness was defined by an increase in stroke volume index (SVI) >15% after volume loading compare to the pre-loading SVI. [file 2047-0525-2-3-S1.pptx]

## Slide 1
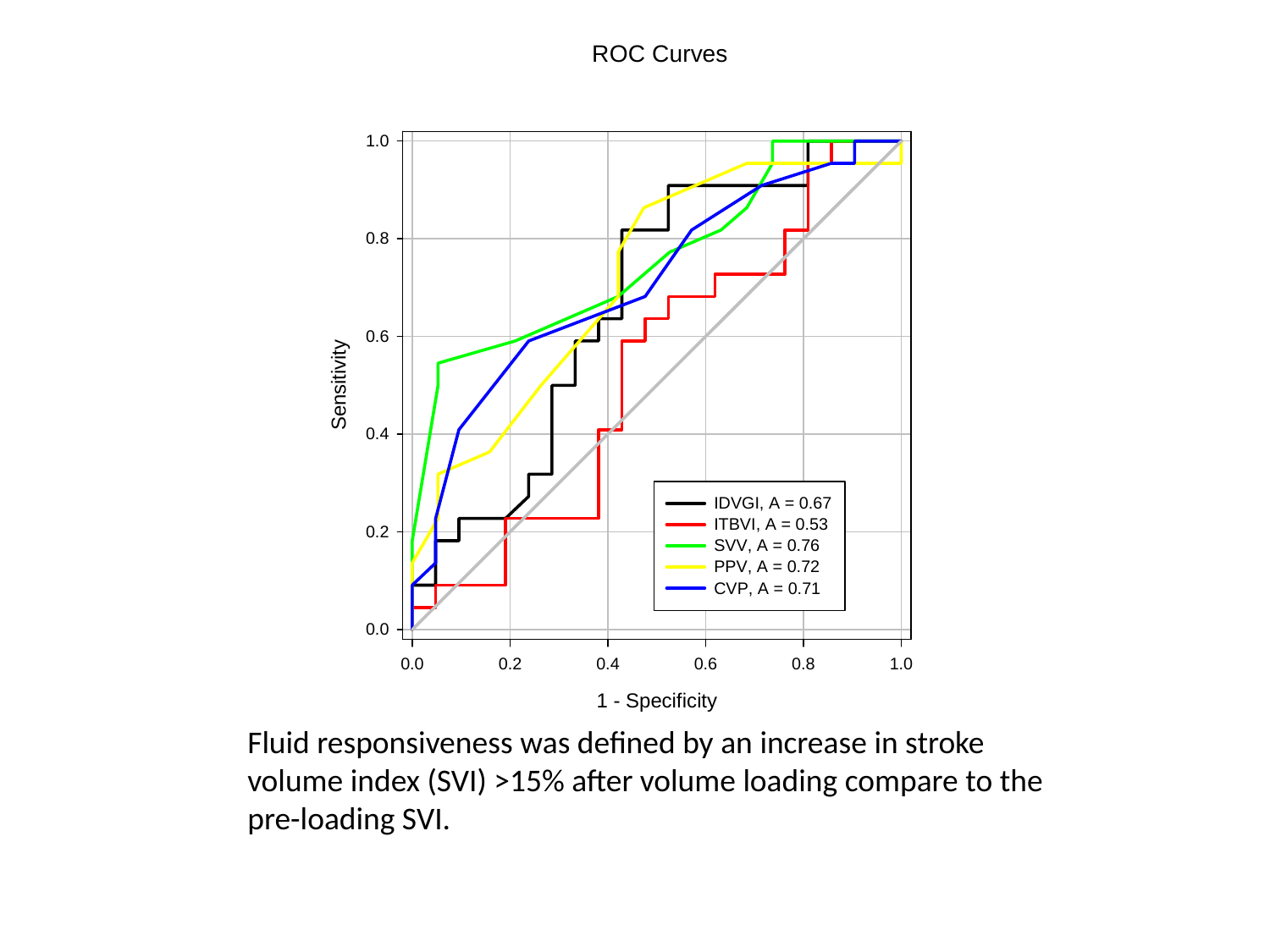

Fluid responsiveness was defined by an increase in stroke volume index (SVI) >15% after volume loading compare to the pre-loading SVI.
